# Improvement of postoperative quality of life in patients with esophageal squamous cell carcinoma: does tea consumption have a role?

**DOI:** 10.1186/s12889-022-14646-z

**Published:** 2022-11-24

**Authors:** Juwei Zhang, Shuang Liu, Jianyu Song, Jinsong Zhou, Qiaoyan Zeng, Zheng Lin, Kaili Yu, Suhong Zhang, Minglian Qiu, Yuanmei Chen, Zhijian Hu

**Affiliations:** 1 Department of Epidemiology and Health Statistics, Fujian Provincial Key Laboratory of Environment Factors and Cancer, School of Public Health, Key Laboratory of Ministry of Education for Gastrointestinal Cancer, Fujian Medical University, Fuzhou, 350122 China; 2 Sun Yat-sen University Cancer Center/Cancer Hospital, Guangzhou, 510060 China; 3 Department of Thoracic Surgery, The First Affiliated Hospital of Fujian Medical University, Fuzhou, 350004 China; 4 Department of Thoracic Surgery, Fujian Provincial Cancer Hospital Affiliation to Fujian Medical University, Fuzhou, 350014 China; 5 Key Laboratory of the Ministry of Education for Gastrointestinal Cancer, Fujian Medical University, Fuzhou, 350122 China

**Keywords:** Esophageal squamous cell carcinoma, Tea consumption, Health-related quality of life, TTD model

## Abstract

**Background:**

To investigate the effect of tea consumption on the improvement of postoperative quality of life in male patients with esophageal squamous cell carcinoma (ESCC).

**Methods:**

The quality of life information of 290 male patients with ESCC was collected. The time to deterioration and the number of events in each area of quality of life was calculated by time-to-deterioration (TTD) model. The association between postoperative tea drinking and postoperative quality of life in male ESCC patients was investigated using the Cox proportional risk model.

**Results:**

Postoperative tea-drinking patients experienced delayed TTD in multiple domains, including general health, physical, role, emotional, and cognitive function, fatigue, nausea and vomiting, dyspnea, loss of appetite, constipation, diarrhea, eating problems, difficulty swallowing, choking while swallowing saliva, dry mouth, taste difficulties, coughing, and speech problems. The multivariate Cox regression analysis showed that drinking tea after surgery improved quality of life, including physical function (HR = 0.722, 95% CI: 0.559-0.933), role function (HR = 0.740, 95% CI: 0.557-0.983), eating problems (HR = 0.718, 95% CI: 0.537-0.960), odynophagia (HR = 0.682, 95% CI: 0.492-0.945), trouble swallowing saliva (HR = 0.624, 95% CI: 0.444-0.877), coughing (HR = 0.627, 95% CI: 0.442-0.889) and speech problems (HR = 0.631, 95% CI: 0.441-0.903). Furthermore, the improvement was more significant in patients who drank tea before surgery and continued to drink tea after surgery.

**Conclusions:**

Postoperative tea drinking had a positive effect on delay in clinical deterioration and improvements in multiple functions and symptoms associated with ESCC in men.

**Supplementary Information:**

The online version contains supplementary material available at 10.1186/s12889-022-14646-z.

## Introduction

According to the 2020 global cancer statistics, the global incidence rate of esophageal cancer is seventh, and the overall mortality rate is sixth, with men having nearly twice the incidence and mortality rate of women [1]. In China, 90% of esophageal cancer is squamous esophageal cancer (ESCC), which has a very poor prognosis and a high mortality rate [2]. Esophagectomy is currently the primary treatment option for patients with esophageal cancer. Surgery improves the five-year survival rate of patients compared to those without surgery [3]. Although surgery and adjuvant therapy can improve patient survival, they also have a significant impact on patients’ quality of life after surgery [4]. As a result, for patients with a poor prognosis and a short life expectancy, optimizing the subsequent quality of life is a critical issue to consider later in order to improve the patient’s well-being during the patient’s remaining life [5].

Tea is gradually becoming one of the most popular beverages in a variety of countries as globalization progresses [6]. Tea is high in flavonoids, alkaloids, phenols, theanine, and other compounds, particularly tea polyphenols, which have antioxidant, anticancer, anti-inflammatory, antibacterial, anti-angiogenic, and apoptotic properties [7]. Tea polyphenols can play an anti-cancer role by scavenging free radicals, blocking lipid peroxidation, and increasing the activity of enzymes in the body [8]. Tea leaf extracts, such as epigallocatechin and theanine, have been shown to inhibit the development and progression of gastrointestinal tumors such as colorectal cancer [9], esophageal cancer [10], and gastric cancer [11] by regulating various signaling pathways [12], initiating apoptosis, inhibiting inflammatory pathways, and suppressing oxidative stress, among other mechanisms. In addition, a report found that tea consumption after gastric cancer surgery could promote the recovery of postoperative gastrointestinal function and improve the quality of life of patients [13]. Meanwhile, tea consumption has been shown to improve cognition, mood, and human brain function [14] and somatic function [15]. All these studies show that drinking tea can improve the health condition of cancer patients. However, no studies have been reported on tea consumption and quality of life in patients with ESCC.

Health-related quality of life (HRQoL) is an important endpoint for measuring cancer patients and survivors [16]. The EORTC QLQ-C30 and EORTC QLQ- OES18 scales were mainly used to evaluate patients’ functional and symptomatic scales in the study of esophageal cancer. In recent years, the deterioration time model in longitudinal analysis has been widely used in the study of quality of life [17]. The time to deterioration (TTD) model is most intuitively defined as the time from randomization in the study to the first deterioration of at least one minimal clinically important difference (MCID) unit compared to the baseline score. Patients who did not deteriorate before exit were reviewed at the last HRQoL assessment [18]. TTD model has been widely used in the evaluation of clinical outcomes because of its advantage of producing more meaningful clinical outcomes. Many studies have used the TTD model to investigate the factors influencing the quality of life in patients with esophageal cancer, but the effect of tea consumption on postoperative quality of life in patients with esophageal cancer is unclear. Therefore, this study investigated the effect of postoperative tea drinking on the postoperative quality of life of male patients with ESCC to provide scientific basis for better improving their quality of life.

In this paper, we based on multiple follow-up questionnaires in male ESCC patients, whose longitudinal quality of life scores were constructed using the TTD model, and the association between postoperative tea consumption and quality of life was evaluated using the Cox proportional hazards model. Subsequently, stratified analysis was used to further explore the relationship between tea consumption and quality of life after preoperative and postoperative tea drinking habits were changed.

## Materials and methods

### Study subjects

Male patients with new-onset ESCC were collected at Fujian Cancer Hospital and the First Affiliated Hospital of Fujian Medical University between December 1, 2014 and March 15, 2021. All enrolled patients were interviewed face-to-face by trained interviewers using standardized questionnaires: the EORTC Quality of Life Questionnaire-Core Questionnaire (EORTC QLQ-C30, version 3.0) and Esophageal Cancer Module (EORTC QLQ- OES18). The inclusion criteria of this study were as follows: (a) patients with radical esophagectomy, (b) ESCC diagnosed by postoperative pathology, and (c) with clear TNM staging, (d) no preoperative chemotherapy, etc. Patients were excluded by the following criteria: (a) patients with other cancers, (b) patients with metastatic tumors or recurrent cases of esophageal cancer, (c) patients with incomplete clinical case information. The tumor stage was determined to be following the American Joint Committee on Cancer Tumor Lymph Node Metastasis (TNM) staging criteria. This study was approved by the Ethics Committee of Fujian Medical University (approval number: 201495). Prior informed consent was obtained from all participants. According to the first follow-up of tea drinking after the operation, the patients were divided into two groups: the tea-drinking group and the non-tea-drinking group.

### Exposure assessment

Tea consumption was defined as drinking pure tea leaves after being brewed or boiled in hot water at least once a week. Participants were asked, “How often do you drink tea?” Participants selected one of the following.” No,” “Less than 5 times per week,” or “Greater than or equal to 5 times per week.” In addition, tea drinkers were asked “What type of tea do you usually drink?”, and were then instructed to select one of four mutually exclusive responses, as follows.“ Unfermented tea (green tea)”, “Semi-fermented tea (oolong tea)”, “Fully fermented tea (including black and dark tea)” or “Other types of tea (flower tea and herbal teas)”. Finally, tea drinkers were also asked how many years they had been drinking this tea, as reported by the patients themselves. We then analyzed the relationship between tea drinking, different types of tea, frequency and duration of tea drinking and quality of life of ESCC patients.

### Follow-up

Patients’ baseline health-related quality of life was assessed within 3 days of hospitalization using the EORTC Quality of Life Questionnaire QLQ-C30 and QLQ-OES18. For postoperative patients, the trained interviewer performed telephone-based follow-up. HRQoL was assessed every 3 months in the first year, every 6 months in the second year, and then annually. Survival time was defined as the time from surgery to death or the end of follow-up.

The EORTC QLQ-C30 has five functional scales (physical, role, emotional, cognitive, social), three symptom scales (fatigue, nausea/vomiting, pain), six individual scales (dyspnea, insomnia, loss of appetite, constipation, diarrhea, economic hardship) and a global health status/quality of life (QOL) scale [19]. The EORTC QLQ-OES18 questionnaire consists of four scales (dysphagia, feeding, reflux, and esophageal pain) and six individual items (swallowing saliva, choking while swallowing, dry mouth, taste problems, cough, and speech problems) [20]. Participants responded to their health status in the week before hospitalization based on questionnaire information.

### Statistical methods

The quality of life scale was calculated using a time to deterioration model with baseline quality of life for all patients included. Time to deterioration was defined as the time between inclusion in the study and the first 5-point decline in quality of life score based on baseline scores or if the patient had not deteriorated prior to this time, reviewed at the last completed quality of life [21]. The TTD calculation process was completed using the QoLR package of R software. The following analyses were completed using SPSS 20.0. The demographic and clinical characteristics of the two groups were compared using chi-square tests, TTD was described using median and quartiles, and the Mann-Whitney test was used to compare the differences in TTD between the two groups. The association of postoperative tea consumption with the EORTC QLQ-C30 and EORTC QLQ-OES18 scales was analyzed using Cox regression models for survival analysis, with relative risks expressed as 95% confidence interval (CI) of the hazard ratio (HR). All statistical tests were two-sided with a significance level of 5%.

## Results

### Follow-up results

Between December 1, 2014 and March 15, 2021, 466 postoperative male patients were enrolled in the study, 176 of whom were eliminated due to lack of partial baseline information, leaving 290 postoperative male ESCC patients enrolled in the study. Evenly distribution of baseline characteristics in the inclusion and exclusion groups (see supplement Table 1). The median follow-up of this study was 35 months (3-72 months), and 77 of the 290 patients died during the study period, with 1-, 3-, and 5-year survival rates of 92.3% (95% CI:0.885-0.949), 76.9% (95% CI:0.708-0.819), and 64.4% (95% CI:0.571- 0.712) (see supplement Table 2).

### Effect of postoperative tea consumption on male postoperative patients with ESCC

#### Demographic and clinical characteristics

In this study, 290 male postoperative patients with ESCC were included, there was no statistical difference in age, chemotherapy, and TNM stage between the two groups of non-tea drinkers and tea drinkers after surgery (*P* > 0.05) (Table 1). In addition, the *p*-value for age was 0.051 for the tea drinking and non-tea drinking groups, therefore, stratified analysis by age, the result indicated that tea consumption had the same effect on quality of life among different age groups (see supplement Table 3 and supplement Table 4).

**Table 1:** Baseline demographic and clinical characteristics of male post-ESCC patients

Variable	Non-tea-drinking [*n* (%)]	Tea-drinking [*n* (%)]	*χ*^*2*^	*P* value
Age (year)			3.818	0.051
< 60	71 (43.6%)	70 (55.1%)		
≥ 60	92 (56.4%)	57 (44.9%)		
Income			1.209	0.272
< 2000	37 (22.7%)	36 (28.3%)		
≥ 2000	126 (77.3%)	91 (71.7%)		
Chemotherapy			0.193	0.660
No	108 (66.3%)	81 (63.8%)		
Yes	55 (33.7%)	46 (36.2%)		
TNM stage			0.692	0.405
I/II stage	87 (53.4%)	74 (58.3%)		
III stage	76 (46.6%)	53 (41.7%)		
Types of tea drinking			–	–
Non-fermented	–	36 (12.4%)		
Semi-fermentation	–	61 (21.0%)		
fully fermented	–	18 (6.2%)		
Other tea	–	12 (4.1%)		
Frequency of tea drinking			–	–
< 5times/week	–	35 (27.6%)		
≥ 5times/week	–	92 (72.4%)		
Duration of tea drinking (year)			–	–
< 30	–	64(53.3%)		
≥ 30	–	56(46.7%)		

#### Baseline quality-of-life scores

Median and quartiles were used to describe baseline quality of life scores. The Mann-Whitney test showed differences between the two groups in the domains of social functioning (*P* = 0.024) and insomnia (*P* = 0.005) on the EORTC QLQ-C30 scale, with no differences in baseline scores for the remaining functional domains (*P* > 0.05); the EORTC QLQ-OES18 scale no differences in baseline scores for all functional domains (*P* > 0.05) (Table 2).

**Table 2:** Preoperative EORTC scale baseline scores in male post-ESCC patients

	Baseline HRQOL scores [*M* (IQR)], *n* = 290			
Domain/scale	Non-tea-drinking	Tea-drinking	*Z*	*P* value
**QLQ-C30**				
Global health status/QOL	75.00(66.67, 83.33)	75.00(66.67, 83.33)	−0.201	0.840
Functional scales				
Physical functioning	100.00(93.33, 100.00)	100.00(93.33, 100.00)	−0.311	0.756
Role functioning	100.00(100.00, 100.00)	100.00(100.00, 100.00)	−0.412	0.680
Emotional functioning	100.00(75.00, 100.00)	100.00(83.33, 100.00)	−1.159	0.246
Cognitive functioning	100.00(100.00, 100.00)	100.00(100.00, 100.00)	−0.148	0.882
Social functioning	66.67(66.67, 100.00)	100.00(66.67, 100.00)	−2.262	0.024
Symptom scales				
Fatigue	0.00(0.00, 22.22)	0.00(0.00, 22.22)	−1.234	0.217
Nausea/vomiting	0.00(0.00, 0.00)	0.00(0.00, 0.00)	−1.029	0.303
Pain	0.00(0.00, 16.67)	0.00(0.00, 16.67)	−1.215	0.224
Dyspnea	0.00(0.00, 0.00)	0.00(0.00, 0.00)	−0.115	0.908
Insomnia	0.00(0.00, 33.33)	0.00(0.00, 0.00)	−2.816	0.005
Appetite loss	0.00(0.00, 33.33)	0.00(0.00, 0.00)	−0.628	0.530
Constipation	0.00(0.00, 0.00)	0.00(0.00, 0.00)	−1.227	0.220
Diarrhea	0.00(0.00, 0.00)	0.00(0.00, 0.00)	−0.177	0.860
**QLQ-QES18**				
General symptom scales				
Dysphagia	77.78(66.67, 88.89)	88.89(66.67，100.00)	−0.547	0.584
Eating problems	0.00(0.00, 6.67)	0.00(0.00, 8.33)	−0.389	0.697
Reflux	0.00(0.00, 0.00)	0.00(0.00, 0.00)	−0.030	0.976
Odynophagia	11.11(0.00, 11.11)	11.11(0.00, 22.22)	−1.155	0.248
General symptom items				
Trouble swallowing saliva	0.00(0.00, 0.00)	0.00(0.00, 0.00)	−0.045	0.964
Choking when swallowing	33.33(0.00, 33.33)	33.33(0.00, 33.33)	−0.526	0.599
Dry mouth	0.00(0.00, 0.00)	0.00(0.00, 0.00)	−1.212	0.225
Trouble with taste	0.00(0.00, 0.00)	0.00(0.00, 0.00)	−0.491	0.624
Coughing	0.00(0.00, 0.00)	0.00(0.00, 0.00)	−0.272	0.786
Speech problems	0.00(0.00, 0.00)	0.00(0.00, 0.00)	−1.288	0.198

#### Time to deterioration of quality of life

Compared to the non-tea drinking group, patients in the postoperative tea drinking group had global health status (*P* = 0.016), physical function (*P* = 0.004), role function (*P* = 0.042), emotional function (*P* = 0.007), cognitive function (*P* = 0.010), fatigue (*P* = 0.032), nausea and vomiting (*P* = 0.015), dyspnea (*P* = 0.040), appetite loss (*P* = 0.017), constipation (*P* = 0.004), diarrhea (*P* = 0.040), eating problems (*P* = 0.006), odynophagia (*P* = 0.001), trouble swallowing saliva (*P* < 0.001), choking when swallowing (*P* = 0.006), dry mouth (*P* = 0.002), trouble with taste (*P* = 0.001), cough (*P* < 0.001) and speech problems (*P* < 0.001) were all delayed in TTD (Table 3). Supplement Table 5 shows the number of patients with deterioration in each domain at each follow-up time point and their percentage of all patients deteriorating in that domain.

**Table 3:** Determination of clinically meaningful time to deterioration in the EORTC QLQ –C30/EORTC QLQ –OES18 scale in two groups of male ESCC patients who do not drink tea and drink tea after surgery

	Time to deterioration [*M* (IQR)], *n* = 290			
Domain/scale	Non-tea-drinking	Tea-drinking	*Z*	*P* value
**QLQ-C30**				
Global health status/QOL	12.48(5.39，23.89)	18(6.37，38.74)	−2.404	0.016
Functional scales				
Physical functioning	12.32(5.75，18.53)	15.47(7.29，31.21)	−2.851	0.004
Role functioning	16.66(10.64，29.60)	22.60(9.90，47.23)	−2.033	0.042
Emotional functioning	17.45(10.64，29.83)	24.97(14.69，45.14)	−2.682	0.007
Cognitive functioning	20.14(12.52，36.76)	24.89(14.68，52.48)	−2.593	0.010
Social functioning	19.29(11.33，29.57)	21.83(9.6，39.06)	−0.326	0.744
Symptom scales				
Fatigue	15.77(7.98，26.38)	21.65(7.69，40.11)	−2.141	0.032
Nausea/vomiting	17.48(10.32，29.60)	25.49(12.58，40.11)	−2.445	0.015
Pain	18.53(11.07，29.90)	24.31(8.51，47.80)	−1.202	0.229
Dyspnea	20.90(12.02，33.48)	25.64(13.24，50.42)	−2.049	0.040
Insomnia	18.53(9.86，36.04)	25.00(13.27，42.35)	−1.942	0.052
Appetite loss	17.18(10.09，29.77)	24.44(13.04，46.36)	−2.378	0.017
Constipation	24.38(15.87，39.79)	30.41(19.78，55.04)	−2.918	0.004
Diarrhea	20.16(11.43，31.92)	24.90(14.00，43.17)	−2.059	0.040
**QLQ-QES18**				
General symptom scales				
Dysphagia	14.65(6.47，22.77)	14.92(7.03，23.52)	−0.551	0.582
Eating problems	15.77(8.48，24.97)	22.01(9.40，42.92)	−2.761	0.006
Reflux	14.55(8.25，23.89)	14.92(7.56，31.11)	−0.859	0.390
Odynophagia	20.11(11.96，29.77)	26.97(15.01，51.77)	−3.214	0.001
General symptom items				
Trouble swallowing saliva	21.88(12.68，35.52)	31.47(18.96，53.78)	−3.668	P < 0.001
Choking when swallowing	21.42(11.47，30.88)	27.70(15.18，53.13)	−2.775	0.006
Dry mouth	22.37(13.47，37.59)	32.43(17.03，59.21)	−3.069	0.002
Trouble with taste	24.97(16.56，40.11)	33.13(20.61，60.47)	−3.182	0.001
Coughing	21.09(10.71，33.48)	27.56(18.07，55.36)	−3.882	P < 0.001
Speech problems	22.37(12.91，37.49)	35.14(18.86，58.66)	−3.752	P < 0.001

#### Association between tea consumption and the EORTC QLQ-C30/EORTC QLQ-OES18 scale

Multivariate Cox regression analysis showed that postoperative tea consumption improved physical function (HR = 0.722, 95% CI: 0.559-0.933), role function (HR = 0.740, 95% CI: 0.557-0.983), eating problems (HR = 0.718, 95% CI: 0.537-0.960), odynophagia (HR = 0.682, 95% CI: 0.492-0.945), trouble swallowing saliva (HR = 0.624, 95% CI: 0.444-0.877), coughing (HR = 0.627, 95% CI: 0.442-0.889) and speech problems (HR = 0.631, 95% CI: 0.441-0.903) (Table 4).

**Table 4:** Association between tea consumption and EORTC QLQ-C30/EORTC QLQ-OES18 scales

Domain/scale	Univariate		Multivariate	
	HR (95% CI)	*P* value	HR (95% CI) ^a^	*P* value
QLQ-C30				
Global health status/QOL	0.790(0.609-1.024)	0.075	0.791(0.609-1.027)	0.078
Physical functioning	0.730(0.567-0.940)	0.015	0.722(0.559-0.933)	0.013
Role functioning	0.713(0.539-0.944)	0.018	0.740(0.557-0.983)	0.037
Emotional functioning	0.816(0.611-1.092)	0.171	0.827(0.618-1.108)	0.203
Cognitive functioning	0.817(0.598-1.117)	0.205	0.812(0.594-1.111)	0.192
Social functioning	1.007(0.755-1.344)	0.962	1.021(0.764-1.364)	0.889
Fatigue	0.866(0.655-1.143)	0.309	0.852(0.643-1.128)	0.262
Nausea/vomiting	0.817(0.605-1.104)	0.188	0.837(0.617-1.134)	0.250
Pain	0.909(0.675-1.224)	0.531	0.894(0.663-1.205)	0.462
Dyspnea	0.819(0.605-1.108)	0.195	0.822(0.606-1.114)	0.206
Insomnia	0.921(0.685-1.238)	0.584	0.911(0.676-1.226)	0.538
Appetite loss	0.862(0.641-1.159)	0.325	0.873(0.648-1.175)	0.370
Constipation	0.740(0.517-1.058)	0.099	0.727(0.507-1.043)	0.084
Diarrhea	0.888(0.651-1.210)	0.452	0.836(0.611-1.143)	0.261
QLQ-QES18				
Dysphagia	0.933(0.715-1.217)	0.610	0.991(0.755-1.302)	0.950
Eating problems	0.717(0.538-0.957)	0.024	0.718(0.537-0.960)	0.026
Reflux	0.958(0741-1.238)	0.742	0.953(0.736-1.235)	0.717
Odynophagia	0.694(0.503-0.958)	0.026	0.682(0.492-0.945)	0.021
Trouble swallowing saliva	0.613(0.437-0.860)	0.005	0.624(0.444-0.877)	0.007
Choking when swallowing	0.762(0.551-1.056)	0.102	0.106(0.549-1.059)	0.106
Dry mouth	0.765(0.550-1.056)	0.113	0.766(0.549-1.069)	0.116
Trouble with taste	0.768(0.525-1.126	0.176	0.744(0.507-1.094)	0.133
Coughing	0.627(0.445-0.882)	0.007	0.627(0.442-0.889)	0.009
Speech problems	0.653(0.459-0.931)	0.018	0.631(0.441-0.903)	0.012

^a^Adjusted for age, income, chemotherapy and TNM stage

Next, we further explored the effects of type, frequency, and duration of tea consumption on the quality of life in ESCC patients. Supplement Table 6 presents the association between different types of tea and quality of life. Unfermented tea plays a positive role in eating problems (HR = 0.584, 95% CI:0.361-0.944), trouble swallowing saliva (HR = 0.553, 95% CI: 0.321-0.950), choking when swallowing (HR = 0.487, 95% CI: 0.279-0.853), coughing (HR = 0.546, 95% CI: 0.312-0.959), and speech problems (HR = 0.490, 95% CI:0.269-0.893). Semi-fermented tea improved physical function (HR = 0.724, 95% CI: 0.525-0.998) and trouble swallowing saliva (HR = 0.621, 95% CI: 0.397-0.971), and observed that odynophagia (HR = 0.176, 95% CI: 0.043-0.720), coughing (HR = 0.291, 95% CI: 0.091-0.928), and speech problems (HR = 0.300 95% CI: 0.094-0.959) could be improved by other types of tea, while no association of fully fermented tea with quality of life was observed.

Table 5 presents the association between the frequency and duration of tea consumption and quality of life in ESCC patients. Drinking tea less than 5 times per week could improve trouble swallowing saliva (HR = 0.359, 95% CI: 0.190-0.677), choking when swallowing (HR = 0.576, 95% CI:0.336-0.989), and coughing (HR = 0.445, 95% CI: 0.247-0.803), and drinking more than 5 times per week had an improving effect on physical function (HR = 0.728, 95% CI:0.549-0.965), role function (HR = 0.686, 95% CI: 0.499-0.943), constipation (HR = 0.597, 95% CI: 0.392-0.911), and speech problems (HR = 0.637, 95% CI: 0.429-0.947). In addition, there were observed for drinking tea for less than 30 years improved odynophagia (HR = 0.0.639, 95% CI: 0.424-0.964), trouble swallowing saliva (HR = 0.615, 95% CI: 0.401-0.942), choking while swallowing (HR = 0.628, 95% CI: 0.410-0.960), coughing (HR = 0.488, 95% CI:0.306-0.779), and speech problems (HR = 0.595, 95% CI: 0.373-0.951). Meanwhile, it was observed that tea drinking for over 30 years could improve trouble swallowing saliva (HR = 0.613, 95% CI: 0.381-0.985) and dry mouth (HR = 0.602, 95% CI: 0.377-0.962).

**Table 5:** Association between frequency and duration of tea consumption and EORTC QLQ-C30/EORTC QLQ-OES18 scales

Domain/scale	Frequency of tea consumption				Duration of tea consumption			
	< 5 times weekly vs. No tea		5 or more times weekly vs. No tea		Less than 30 years vs. No tea		30 or more than 30 years vs. No tea	
	HR (95% CI) ^a^	*P* value	HR (95% CI) ^a^	*P* value	HR (95% CI) ^a^	*P* value	HR (95% CI) ^a^	*P* value
QLQ-C30								
Global health status/QOL	0.683(0.451-1.036)	0.073	0.839(0.629-1.118)	0.230	0.756(0.541-1.057)	0.101	0.877(0.627-1.226)	0.442
Physical functioning	0.708(0.476-1.053)	0.088	0.728(0.549-0.965)	0.027	0.742(0.534-1.029)	0.074	0.735(0.530-1.021)	0.066
Role functioning	0.899(0.583-1.386)	0.630	0.686(0.499-0.943)	0.020	0.707(0.486-1.029)	0.070	0.766(0.533-1.101)	0.150
Emotional functioning	0.746(0.474-1.175)	0.206	0.864(0.626-1.193)	0.375	0.779(0.538-1.129)	0.187	0.836(0.571-1.225)	0.359
Cognitive functioning	0.770(0.473-1.254)	0.291	0.830(0.588-1.172)	0.290	0.786(0.530-1.166)	0.232	0.756(0.495-1.154)	0.195
Social functioning	1.165(0.763-1.779)	0.480	0.962(0.693-1.335)	0.816	0.969(0.670-1.400)	0.866	1.207(0.832-1.750)	0.322
Fatigue	0.774(0.499-1.201)	0.254	0.885(0.650-1.203)	0.435	0.844(0.590-1.207)	0.352	0.931(0.651-1.331)	0.696
Nausea/vomiting	0.850(0.538-1.342)	0.485	0.831(0.592-1.166)	0.285	0.748(0.508-1.100)	0.140	1.007(0.680-1.491)	0.973
Pain	0.893(0.566-1.409)	0.628	0.894(0.642-1.245)	0.508	0.925(0.637-1.343)	0.681	0.855(0.572-1.279)	0.446
Dyspnea	0.639(0.385-1.060)	0.083	0.903(0.649-1.256)	0.544	0.746(0.505-1.103)	0.142	0.952(0.641-1.413)	0.806
Insomnia	0.658(0.402-1.077)	0.096	1.037(0.751-1.432)	0.827	0.838(0.574-1.225)	0.362	1.009(0.687-1.482)	0.962
Appetite loss	0.745(0.469-1.185)	0.214	0.935(0.673-1.297)	0.686	0.887(0.616-1.277)	0.520	0.871(0.582-1.302)	0.500
Constipation	1.098(0.667-1.809)	0.714	0.597(0.392-0.911)	0.017	0.724(0.459-1.141)	0.164	0.705(0.432-1.148)	0.160
Diarrhea	0.680(0.413-1.118)	0.128	0.911(0.647-1.283)	0.592	0.722(0.486-1.073)	0.107	0.957(0.638-1.434)	0.830
QLQ-QES18								
Dysphagia	0.678(0.431-1.065)	0.092	1.153(0.860-1.547)	0.342	0.977(0.694-1.375)	0.893	0.980(0.684-1.404)	0.911
Eating problems	0.650(0.414-1.021)	0.062	0.750(0.544-1.035)	0.080	0.794(0.549-1.148)	0.220	0.699(0.478-1.023)	0.065
Reflux	0.748(0.495-1.128)	0.166	1.057(0.796-1.403)	0.702	0.999(0.722-1.382)	0.994	0.956(0.682-1.341)	0.795
Odynophagia	0.640(0.387-1.057)	0.081	0.701(0.488-1.006)	0.054	0.639(0.424-0.964)	0.033	0.709(0.456-1.102)	0.127
Trouble swallowing saliva	0.359(0.190-0.677)	0.002	0.760(0.528-1.094)	0.140	0.615(0.401-0.942)	0.026	0.613(0.381-0.985)	0.043
Choking when swallowing	0.576(0.336-0.989)	0.045	0.852(0.596-1.218)	0.380	0.628(0.410-0.960)	0.032	0.919(0.603-1.401)	0.696
Dry mouth	0.658(0.392-1.106)	0.114	0.818(0.566-1.181)	0.283	0.905(0.603-1.356)	0.627	0.602(0.377-0.962)	0.034
Trouble with taste	0.597(0.320-1.116)	0.106	0.812(0.534-1.236)	0.331	0.792(0.493-1.274)	0.337	0.671(0.396-1.138)	0.139
Coughing	0.445(0.247-0.803)	0.007	0.719(0.492-1.052)	0.090	0.488(0.306-0.779)	0.003	0.812(0.523-1.261)	0.354
Speech problems	0.613(0.345-1.091)	0.096	0.637(0.429-0.947)	0.026	0.595(0.373-0.951)	0.030	0.694(0.440-1.094)	0.116

^a^Controlling for demographic and clinical characteristics variables: age, income, Chemotherapy and TNM stage

#### Association between preoperative and postoperative changes in tea drinking habits and quality of life in 290 male patients with esophageal squamous carcinoma

The above results suggest that postoperative tea drinking can improve multiple scales of quality of life in male ESCC postoperative patients, but many patients’ preoperative and postoperative tea drinking habits change, and what effect the change will have on patients’ quality of life we do not know. Therefore, we stratified 290 patients by preoperative tea consumption to further investigate the association between preoperative and postoperative changes in tea drinking habits and quality of life. Among them, 65 were preoperative non-tea drinkers and 225 were tea drinkers.

#### Association between change in tea drinking habits and quality of life after surgery in 65 patients who did not drink tea before surgery

The results showed that the median TTD in the postoperative tea drinking group was 40.94 months [IQR: 21.77, 64.10] compared with the median TTD of 22.57 months [IQR: 11.47, 36.76] in the non-tea-drinking group, and the TTD in the cough domain was delayed (*P* = 0.034) (Supplement Table 7).

The association between tea consumption and the EORTC QLQ-C30/EORTC QLQ-OES18 scale in 65 male ESCC patients was analyzed using a multivariate Cox regression model, and no association was found (*P* > 0.05) (Supplement Table 8).

#### Association between change in tea drinking habits and quality of life after surgery in 225 patients who drank tea before surgery

Compared to the non-tea drinking group, patients in the postoperative tea drinking group had better global health status (*P* = 0.018), physical function (*P* = 0.006), role function (*P* = 0.012), emotional function (*P* = 0.004) cognitive function (*P* = 0.013), insomnia (*P* = 0.026), appetite loss (*P* = 0.019), constipation (*P* = 0.013), eating problems (*P* = 0.013), odynophagia (*P* = 0.002), trouble swallowing saliva (*P* < 0.001), and choking when swallowing (*P* = 0.003), dry mouth (*P* = 0.004), trouble with taste (*P* = 0.001), cough (*P* = 0.001) and speech problems (*P* < 0.001) were all delayed in TTD (Supplement Table 9).

Multivariate Cox regression analysis showed that postoperative tea consumption improved global health status (*HR* = 0.725, 95%*CI*: 0.541-0.972), physical function (*HR* = 0.689, *95%* CI: 0.518-0.917), role function (*HR* = 0.678, *95%* CI: 0.493-0.933), eating problems (*HR* = 0.701, *95%* CI: 0.502-0.980), odynophagia (*HR* = 0.679, *95%* CI: 0.469-0.983), trouble swallowing saliva (*HR* = 0.596, *95%* CI: 0.407-0.872), cough (*HR* = 0.615, *95%* CI: 0.417-0.907), and speech problems (*HR* = 0.556, *95%* CI: 0.372-0.830) (Supplement Table 10).

## Discussion

In the clinical setting, physicians and families hope to improve ESCC patients’ survival through surgery and postoperative adjuvant therapy, but they rarely seek to improve patients’ quality of life after surgery. Fortunately, an increasing number of researchers have recognized the importance of improving the quality of life for patients with a poor prognosis or those who are already in the advanced stages of cancer for the remainder of their lives. In this study, we collected information on patients’ postoperative lifestyle habits and postoperative health-related quality of life. We first analyzed the association study of preoperative tea consumption with postoperative quality of life in male ESCC patients and did not find an association. Therefore, we analyzed the longitudinal effect of postoperative tea consumption on the quality of life of male esophageal squamous cell carcinoma patients using the TTD model, which facilitates both the comprehensive assessment of the effect of tea consumption on patients’ quality of life and the interpretation of the results by clinicians to make scientific recommendations. Our results show that patients who consumed tea postoperatively had delayed TTD in multiple domains assessed by the EORTC QLQ-C30/EORTC QLQ-OES18, including global health status, physical function, role function, emotional function, cognitive function, fatigue, nausea and vomiting, dyspnea, appetite loss, constipation, diarrhea, eating problems, odynophagia, trouble swallowing saliva, choking when swallowing, dry mouth, trouble with taste, cough, and speech problems. The results of multivariate Cox regression analysis showed that postoperative tea consumption improved quality of life, including physical function, role function, eating problems, odynophagia, trouble swallowing saliva, cough and speech problems. Furthermore, in addition to fully fermented teas, different types of tea have different aspects of improvement in the quality of life of ESCC patients, and the improvement of symptoms is particularly evident. Also, the frequency and duration of tea consumption had a positive effect on some of the postoperative functions and symptoms of patients. In conclusion, this study supports the hypothesis that postoperative tea consumption is beneficial for men with esophageal squamous cell carcinoma, regardless of preoperative tea consumption, and that long-term tea consumption results in more significant improvements in quality of life.

Currently, an increasing number of studies have confirmed that drinking tea can promote health through its active ingredients. According to Zafar Rasheed, there is molecular evidence that black teas can prevent tumor development and inhibit key events in cancer stimulation, lowering the risk of cancer development and progression [22]. Our data showed that long-term tea drinkers had a delayed time to deterioration in global health status, physical function, role function, emotional function, and cognitive function, with a significant improvement in physical and role function in particular. Previous research has found that theaflavins in tea can improve memory impairment and depression-like behavior via modulating microglia activation, as well as decrease neuroinflammation and avoid symptoms of inflammation-related brain illnesses [23]. The brain has an important role in the maintenance of somatic, role, emotional, and cognitive functions; therefore, it can be hypothesized that the active substances in tea can improve patients’ postoperative emotional and cognitive functions by maintaining human brain functions.

For the EORTC QLQ-OES18 scale, the analysis found that postoperative tea consumption improved significantly on eating problems, odynophagia, trouble swallowing saliva, coughing and speech problems. The main mechanism may be through the antioxidant and anti-inflammatory effects of L-theanine [24] and epigallocatechin [25] components in tea to achieve improve pain and maintain a stable oral environment. This suggests that cancer patients may improve their oral environment and thus symptoms such as difficulty eating, dry mouth, cough and swallowing by drinking tea. Simultaneously, Timothy Bond et colleagues. Discovered that drinking tea can assist ameliorate intestinal ecological dysbiosis by regulating the distribution of the intestinal microbiota [26]. Therefore, we speculate that the improvement of constipation and diarrhea may be related to this mechanism. Reflux is a relatively common symptom in patients with esophageal cancer after surgery [27], but no association was found between postoperative tea consumption and reflux in this study, which is consistent with the findings of Cao et al. [28]. In conclusion, we can speculate that tea drinking mainly improves the problems of eating and swallowing in postoperative esophageal cancer patients, and does not provide significant relief for symptoms such as reflux and nausea and vomiting.

In addition, we considered that most patients with ESCC were male and most of them had tea drinking habits before surgery, and patients and their family members might ask whether they could drink tea after surgery and how tea drinking would affect their subsequent recovery. Analysis after stratification of patients according to their preoperative tea consumption revealed a delay in TTD of cough in patients who consumed tea postoperatively compared to those who did not consume tea postoperatively. However, among patients who drank tea preoperatively, those who continued to drink tea postoperatively had delayed TTD for global health, physical, emotional and cognitive function, insomnia, appetite loss, constipation, eating problems, odynophagia, trouble swallowing saliva, choking when swallowing, dry mouth, trouble with taste, cough and speech problems. And tea consumption improved global health, physical function, role function, eating problems, odynophagia, trouble swallowing saliva, cough, and speech problems. In conclusion, the findings of this study were that postoperative tea consumption had an improvement in several functional neighborhoods in men with esophageal squamous carcinoma regardless of preoperative tea consumption, and the improvement was more pronounced in patients who had been drinking tea preoperatively and postoperatively.

HRQoL is usually evaluated at different times in clinical trials to analyze HRQoL dynamics and to comprehensively assess the impact of treatment on patient HRQoL levels, so it makes sense to perform a longitudinal analysis of HRQoL [29]. The TTD model is a method of longitudinal analysis and is well suited for evaluating factors affecting the quality of life of cancer patients because its results are more easily understood by clinicians [30]. Therefore, the use of the TTD model is one of the strengths of this study. The second advantage is to study the effect of preoperative and postoperative changes in tea drinking habits on quality of life from a dynamic perspective, providing scientific guidance to improve the quality of life of male ESCC patients after surgery. However, there are some limitations, firstly, the subjects of this study were only for the male group and could not provide useful suggestions for the female group; Second, the absence of some information from some patients led to the inclusion of fewer study subjects. Future studies should increase the sample size, obtain more detailed information from hospitals and patients as much as possible, and consider exploring the relationship between postoperative tea consumption and quality of life in the female population when the sample size allows, so as to improve the findings.

## Conclusions

Postoperative tea consumption improved functional performance and delayed deterioration of global health status, physical, role, and cognitive function, as well as odynophagia, bowel, choking when swallowing, and speech problems in male patients with ESCC. Furthermore, the improvement in quality of life of patients who drank tea before and after surgery was more significant.

## Supplementary Information

**Additional file 1 Supplement Table 1.** Baseline characteristics of the inclusion and exclusion groups. **Supplement Table 2.** Follow-up results of the EORTC QLQ-C30 and EORTC QLQ-OES18 scales in 290 male patients with ESCC. **Supplement Table 3.** Cox regression analysis for evaluating the association between tea consumption and EORTC QLQ-C30/EORTC QLQ-OES18 scales in age < 60 patients. **Supplement Table 4.** Cox regression analysis for evaluating the association between tea consumption and EORTC QLQ-C30/EORTC QLQ-OES18 scales in age ≥ 60 patients. **Supplement Table 5.** The number of patients with deterioration in each domain at each follow-up time point and their percentage of all patients deteriorating in that domain. **Supplement Table 6.** Association between types of tea consumption and EORTC QLQ-C30/EORTC QLQ-OES18 scales. **Supplement Table 7.** 65 male ESCC patients who did not drink tea preoperatively, and the EORTC QLQ -C30/EORTC QLQ -OES18 EORTC scale for both non-tea and tea drinking groups postoperatively to determine the clinically meaningful time to deterioration. **Supplement Table 8.** Association between tea consumption and EORTC QLQ-C30/EORTC QLQ-OES18 scales in 65 male ESCC patients. **Supplement Table 9.** 225 male ESCC patients who consumed tea preoperatively, time to clinically significant deterioration determined by the EORTC QLQ -C30/EORTC QLQ -OES18 preoperative EORTC scale in both the no-tea and tea groups postoperatively. **Supplement Table 10.** Association between tea consumption and EORTC QLQ-C30/EORTC QLQ-OES18 scales in 225 male ESCC patients.

## Data Availability

The datasets generated during and/or analysed during the current study are available from the corresponding author on reasonable request.

## Abbreviations

ESCC: Esophageal squamous cell carcinoma
HRQoL: Health-related quality of life
TTD: The time to deterioration
MCID: Minimal clinically important difference
TNM: Tumor Lymph Node Metastasis
HR: Hazard ratio
95% CI: 95% confidence interval
