# Supplementary material for: Improvement of postoperative quality of life in patients with esophageal squamous cell carcinoma: does tea consumption have a role?
Source: BMC Public Health. 2022 Nov 24;22:2165. doi: 10.1186/s12889-022-14646-z (PMC9694859; doi:10.1186/s12889-022-14646-z)
Supplement: Supplementary file 1 — Additional file 1 Supplement Table 1. Baseline characteristics of the inclusion and exclusion groups. Supplement Table 2. Follow-up results of the EORTC QLQ-C30 and EORTC QLQ-OES18 scales in 290 male patients with ESCC. Supplement Table 3. Cox regression analysis for evaluating the association between tea consumption and EORTC QLQ-C30/EORTC QLQ-OES18 scales in age < 60 patients. Supplement Table 4. Cox regression analysis for evaluating the association between tea consumption and EORTC QLQ-C30/EORTC QLQ-OES18 scales in age ≥ 60 patients. Supplement Table 5. The number of patients with deterioration in each domain at each follow-up time point and their percentage of all patients deteriorating in that domain. Supplement Table 6. Association between types of tea consumption and EORTC QLQ-C30/EORTC QLQ-OES18 scales. Supplement Table 7. 65 male ESCC patients who did not drink tea preoperatively, and the EORTC QLQ -C30/EORTC QLQ -OES18 EORTC scale for both non-tea and tea drinking groups postoperatively to determine the clinically meaningful time to deterioration. Supplement Table 8. Association between tea consumption and EORTC QLQ-C30/EORTC QLQ-OES18 scales in 65 male ESCC patients. Supplement Table 9. 225 male ESCC patients who consumed tea preoperatively, time to clinically significant deterioration determined by the EORTC QLQ -C30/EORTC QLQ -OES18 preoperative EORTC scale in both the no-tea and tea groups postoperatively. Supplement Table 10. Association between tea consumption and EORTC QLQ-C30/EORTC QLQ-OES18 scales in 225 male ESCC patients. [file 12889_2022_14646_MOESM1_ESM.docx]

**Supplement table**

**Supplement table 1.** Baseline characteristics of the inclusion and exclusion groups.

| Variable |  | Exclusion [n (%)] |  | Inclusion [n (%)] |  | *χ^2^* |  | *P* |
| --- | --- | --- | --- | --- | --- | --- | --- | --- |
| Age(year) |  |  |  |  |  | 0.440 |  | 0.507 |
| <60 |  | 80 (45.5%) |  | 141 (48.6%) |  |  |  |  |
| ≥60 |  | 96 (54.5%) |  | 149 (51.4%) |  |  |  |  |
| Income |  |  |  |  |  | 0.745 |  | 0.388 |
| <60 |  | 46(28.9%) |  | 73(25.2%) |  |  |  |  |
| ≥60 |  | 113(71.1%) |  | 217（74.8） |  |  |  |  |
| Types of tea drinking |  |  |  |  |  | 5.447 |  | 0.244 |
| Non-tea-drinking |  | 98(64.9%) |  | 163(56.2%) |  |  |  |  |
| Non-fermented |  | 13(8.8%) |  | 36(12.4%) |  |  |  |  |
| Semi-fermentation |  | 21(13.9%) |  | 61(21.0%) |  |  |  |  |
| fully fermented |  | 8(5.3%) |  | 18(6.2%) |  |  |  |  |
| Other tea |  | 7(4.6%) |  | 12(4.1%) |  |  |  |  |
| Frequency of tea drinking |  |  |  |  |  | 1.707 |  | 0.426 |
| Non-tea-drinking |  | 98(62.4%) |  | 163(56.2%) |  |  |  |  |
| 1-5times/week |  | 15(9.6%) |  | 35(12.1%) |  |  |  |  |
| ≥5times/week |  | 44(28.0%) |  | 92(31.7%) |  |  |  |  |
| Duration of drinking(year) |  |  |  |  |  | 4.053 |  | 0.132 |
| Non-tea-drinking |  | 98(67.6%) |  | 163（61.0%） |  |  |  |  |
| <10 |  | 5(3.4%) |  | 14(4.9%) |  |  |  |  |
| ≥10 |  | 42(29.0%) |  | 106(37.5%) |  |  |  |  |

**Supplement table 2**. Follow-up results of the EORTC QLQ-C30 and EORTC QLQ-OES18 scales in 290 male patients with ESCC

| Follow-up times |  | Time to baseline follow-up (months) |  | Number of subjects |  | Follow-up rate |
| --- | --- | --- | --- | --- | --- | --- |
| baseline |  | 0 |  | 290 |  | 100% |
| 1st |  | 3 |  | 257 |  | 88.62% |
| 2nd |  | 6 |  | 238 |  | 82.07% |
| 3rd |  | 9 |  | 204 |  | 70.34% |
| 4th |  | 12 |  | 190 |  | 65.52% |
| 5th |  | 18 |  | 164 |  | 56.55% |
| 6th |  | 24 |  | 130 |  | 44.38% |
| 7th |  | 30 |  | 120 |  | 41.38% |
| 8th |  | 36 |  | 106 |  | 36.55% |
| 9th |  | 42 |  | 95 |  | 32.76% |
| 10th |  | 48 |  | 76 |  | 26.21% |
| 11th |  | 54 |  | 66 |  | 22.76% |
| 12th |  | 60 |  | 54 |  | 18.62% |
| 13th |  | 66 |  | 27 |  | 9.31% |
| 14th |  | 72 |  | 13 |  | 4.48% |

**Supplement table 3.** Cox regression analysis for evaluating the association between tea consumption and EORTC QLQ-C30/EORTC QLQ-OES18 scales in age <60 patients.

| Domain/scale |  | Univariate | | |  | Multivariate | | |
| --- | --- | --- | --- | --- | --- | --- | --- | --- |
|  |  | HR (95% CI) |  | P value |  | HR (95% CI) * |  | P value |
| QLQ-C30 |  |  |  |  |  |  |  |  |
| Global health status/QOL |  | 0.911(0.628-1.321) |  | 0.622 |  | 0.909(0.627-1.319) |  | 0.615 |
| Physical functioning |  | 0.650(0.454-0.930) |  | 0.018 |  | 0.650(0.453-0.932) |  | 0.019 |
| Role functioning |  | 0.651(0.431-0.984) |  | 0.042 |  | 0.680(0.449-1.030) |  | 0.690 |
| Emotional functioning |  | 1.029(0.678-1.561) |  | 0.895 |  | 1.048(0.699-1.593) |  | 0.827 |
| Cognitive functioning |  | 1.022(0.670-1.558) |  | 0.921 |  | 1.031(0.675-1.575) |  | 0.887 |
| Social functioning |  | 1.253(0.836-1.877) |  | 0.275 |  | 1.248(0.831-1.873) |  | 0.285 |
| Fatigue |  | 1.057(0.716-1.562) |  | 0.779 |  | 1.061(0.718-1.569) |  | 0.765 |
| Nausea/vomiting |  | 0.790(0.510-1.224) |  | 0.291 |  | 0.799(0.514-1.240) |  | 0.317 |
| Pain |  | 1.030(0.686-1.546) |  | 0.888 |  | 1.040(0.692-1.562) |  | 0.850 |
| Dyspnea |  | 0.931(0.601-1.441) |  | 0.747 |  | 0.942(0.607-1.462) |  | 0.790 |
| Insomnia |  | 0.895(0.596-1.345) |  | 0.594 |  | 0.909(0.604-1.366) |  | 0.645 |
| Appetite loss |  | 0.984(0.644-1.504) |  | 0.942 |  | 1.028(0.672-1.575) |  | 0.897 |
| Constipation |  | 0.887(0.539-1.462) |  | 0.639 |  | 0.887(0.538-1.465) |  | 0.640 |
| Diarrhea |  | 0.819(0.542-1.236) |  | 0.341 |  | 0.801(0.529-1.213) |  | 0.295 |
| QLQ-QES18 |  |  |  |  |  |  |  |  |
| Dysphagia |  | 0.740(0.508-1.080) |  | 0.119 |  | 0.785(0.535-1.150) |  | 0.214 |
| Eating problems |  | 0.864(0.569-1.313) |  | 0.495 |  | 0.890(0.583-1.359) |  | 0.589 |
| Reflux |  | 0.933(0.649-1.340) |  | 0.706 |  | 0.938(0.652-1.349) |  | 0.730 |
| Odynophagia |  | 0.686(0.444-1.058) |  | 0.088 |  | 0.692(0.448-1.070) |  | 0.098 |
| Trouble swallowing saliva |  | 0.695(0.436-1.108) |  | 0.126 |  | 0.708(0.443-1.131) |  | 0.148 |
| Choking when swallowing |  | 0.817(0.518-1.287) |  | 0.383 |  | 0.828(0.524-1.308) |  | 0.418 |
| Dry mouth |  | 0.893(0.567-1.408) |  | 0.627 |  | 0.886(0.560-1.401) |  | 0.604 |
| Trouble with taste |  | 0.910(0.547-1.514) |  | 0.717 |  | 0.920(0.551-1.536) |  | 0.750 |
| Coughing |  | 0.480(0.297-0.776) |  | 0.003 |  | 0.491(0.303-0.797) |  | 0.004 |
| Speech problems |  | 0.574(0.355-0.928) |  | 0.024 |  | 0.574(0.353-0.932) |  | 0.025 |

*Adjusting for income, chemotherapy and TNM stage.

**Supplement table 4.** Cox regression analysis for evaluating the association between tea consumption and EORTC QLQ-C30/EORTC QLQ-OES18 scales in age≥60 patients.

| Domain/scale |  | Univariate | | |  | Multivariate | | |
| --- | --- | --- | --- | --- | --- | --- | --- | --- |
|  |  | HR (95% CI) |  | P value |  | HR (95% CI) * |  | P value |
| QLQ-C30 |  |  |  |  |  |  |  |  |
| Global health status/QOL |  | 0.697(0.481-1.010) |  | 0.057 |  | 0.692(0.477-1.004) |  | 0.053 |
| Physical functioning |  | 0.800(0.557-1.147) |  | 0.225 |  | 0.795(0.554-1.140) |  | 0.212 |
| Role functioning |  | 0.812(0.553-1.193) |  | 0.289 |  | 0.822(0.558-1.210) |  | 0.320 |
| Emotional functioning |  | 0.635(0.416-0.970) |  | 0.036 |  | 0.644(0.421-0.984) |  | 0.042 |
| Cognitive functioning |  | 0.600(0.372-0.968) |  | 0.036 |  | 0.602(0.373-0.971) |  | 0.038 |
| Social functioning |  | 0.778(0.509-1.189) |  | 0.245 |  | 0.789(0.516-1.207) |  | 0.274 |
| Fatigue |  | 0.702(0.467-1.056) |  | 0.089 |  | 0.702(0.467-1.056) |  | 0.090 |
| Nausea/vomiting |  | 0.875(0.576-1.330) |  | 0.532 |  | 0.885(0.581-1.347) |  | 0.568 |
| Pain |  | 0.777(0.498-1.214) |  | 0.268 |  | 0.769(0.492-1.202) |  | 0.249 |
| Dyspnea |  | 0.709(0.462-1.088) |  | 0.115 |  | 0.710(0.462-1.090) |  | 0.118 |
| Insomnia |  | 0.908(0.588-1.403) |  | 0.665 |  | 0.905(0.586-1.398) |  | 0.654 |
| Appetite loss |  | 0.762(0.501-1.159) |  | 0.204 |  | 0.770(0.505-1.174) |  | 0.225 |
| Constipation |  | 0.616(0.363-1.046) |  | 0.073 |  | 0.610(0.359-1.037) |  | 0.068 |
| Diarrhea |  | 0.865(0.536-1.394) |  | 0.551 |  | 0.872(0.541-1.407) |  | 0.575 |
| QLQ-QES18 |  |  |  |  |  |  |  |  |
| Dysphagia |  | 1.185(0.812-1.728) |  | 0.379 |  | 1.290(0.881-1.891) |  | 0.191 |
| Eating problems |  | 0.603(0.402-0.903) |  | 0.014 |  | 0.602(0.401-0.903) |  | 0.014 |
| Reflux |  | 0.966(0.668-1.397) |  | 0.854 |  | 0.978(0.675-1.416) |  | 0.906 |
| Odynophagia |  | 0.644(0.393-1.057) |  | 0.082 |  | 0.667(0.406-1.094) |  | 0.109 |
| Trouble swallowing saliva |  | 0.532(0.322-0.878) |  | 0.014 |  | 0.541(0.327-0.893) |  | 0.016 |
| Choking when swallowing |  | 0.685(0.426-1.103) |  | 0.120 |  | 0.691(0.429-1.113) |  | 0.129 |
| Dry mouth |  | 0.630(0.385-1.032) |  | 0.067 |  | 0.635(0.387-1.042) |  | 0.073 |
| Trouble with taste |  | 0.574(0.314-1.049) |  | 0.071 |  | 0.569(0.311-1.042) |  | 0.068 |
| Coughing |  | 0.796(0.488-1.297) |  | 0.360 |  | 0.819(0.501-1.337) |  | 0.424 |
| Speech problems |  | 0.708(0.418-1.200) |  | 0.200 |  | 0.698(0.412-1.184) |  | 0.182 |

*Adjusting for income, chemotherapy and TNM stage.

**Supplement table 5.** the number of patients with deterioration in each domain at each follow-up time point and their percentage of all patients deteriorating in that domain.

| domains | Follow-up times | | | | | | | | | | | | | |
| --- | --- | --- | --- | --- | --- | --- | --- | --- | --- | --- | --- | --- | --- | --- |
|  | 1st | 2nd | 3rd | 4th | 5th | 6th | 7th | 8th | 9th | 10th | 11th | 12th | 13th | 14th |
| QLQ-C30 |  |  |  |  |  |  |  |  |  |  |  |  |  |  |
| Global health status/QOL | 89(38.03) | 40(17.09) | 32(13.68) | 15(6.41) | 20(8.55) | 12(5.13) | 8(3.42) | 12(5.13) | 2(0.85) | 3(1.28) | 1(0.43) | 0(0.00) | 0(0.00) | 0(0.00) |
| Physical functioning | 75(30.00) | 56(22.40) | 36(14.40) | 27(10.80) | 17(6.80) | 10(4.00) | 10(4.00) | 12(4.80) | 2(0.80) | 2(0.80) | 1(0.40) | 0(0.00) | 2(0.80) | 0(0.00) |
| Role functioning | 47(22.82) | 40(19.42) | 36(17.48) | 24(11.65) | 20(9.71) | 5(2.43) | 11(5.34) | 15(7.28) | 3(1.46) | 1(0.49) | 11(5.34) | 12(5.83) | 1(0.49) | 0(0.00) |
| Emotional functioning | 29(15.59) | 36(19.35) | 37(19.89) | 15(8.06) | 20(10.75) | 17(9.14) | 6(3.23) | 15(8.06) | 5(2.69) | 1(0.54) | 1(0.54) | 3(1.61) | 1(0.54) | 0(0.00) |
| Cognitive functioning | 33(20.37) | 24(14.81) | 28(17.28) | 19(11.73) | 13(8.02) | 3(1.85) | 9(5.56) | 16(9.88) | 7(4.32) | 1(0.62) | 6(3.70) | 3(1.85) | 0(0.00) | 0(0.00) |
| Social functioning | 41(21.81) | 36(19.15) | 24(12.77) | 18(9.57) | 14(7.45) | 14(7.45) | 11(5.85) | 13(6.91) | 5(2.66) | 10(5.32) | 2(1.06) | 5(2.66) | 2(1.06) | 0(0.00) |
| Fatigue | 61(30.05) | 40(19.70) | 32(15.76) | 16(7.88) | 16(7.88) | 10(4.93) | 11(5.42) | 10(4.93) | 1(0.49) | 1(0.49) | 2(0.99) | 1(0.49) | 0(0.00) | 2(0.99) |
| Nausea/vomiting | 46(26.29) | 26(14.86) | 30(17.14) | 15(8.57) | 16(9.14) | 13(7.43) | 12(6.86) | 10(5.71) | 1(0.57) | 1(0.57) | 2(1.14) | 1(0.57) | 0(0.00) | 2(1.14) |
| Pain | 50(27.93) | 34(18.99) | 27(15.08) | 14(7.82) | 14(7.82) | 13(7.26) | 7(3.91) | 11(6.15) | 3(1.68) | 2(1.12) | 2(1.12) | 1(0.56) | 0(0.00) | 1(0.56) |
| Dyspnea | 30(17.24) | 37(21.26) | 28(16.09) | 17(9.77) | 18(1034) | 12(6.90) | 11(6.32) | 10(5.75) | 5(2.87) | 3(1.72) | 1(0.57) | 1(0.57) | 0(0.00) | 1(0.57) |
| Insomnia | 39(21.91) | 35(19.66) | 22(12.36) | 19(10.67) | 18(10.11) | 11(6.18) | 12(6.74) | 9(5.06) | 3(1.69) | 5(2.81) | 3(1.69) | 1(0.56) | 1(0.56) | 0(0.00) |
| Appetite loss | 37(20.67) | 38(21.23) | 33(18.44) | 15(8.38) | 15(8.38) | 12(6.70) | 6(3.35) | 15(8.38) | 4(2.23) | 0(0.00) | 1(0.56) | 2(1.12) | 1(0.56) | 0(0.00) |
| Constipation | 19(15.32) | 16(12.90) | 21(16.94) | 14(11.29) | 15(12.10) | 9(7.26) | 11(8.87) | 10(8.06) | 4(3.23) | 0(0.00) | 2(1.61) | 3(2.42) | 0(0.00) | 0(0.00) |
| Diarrhea | 35(21.47) | 24(14.72) | 36(22.09) | 14(8.59) | 15(9.20) | 10(6.13) | 10(6.13) | 13(7.98) | 1(0.61) | 0(0.00) | 1(0.61) | 3(1.84) | 1(0.61) | 0(0.00) |
| QLQ-QES18 |  |  |  |  |  |  |  |  |  |  |  |  |  |  |
| Dysphagia | 74(33.33) | 41(18.47) | 45(20.27) | 38(17.12) | 9(4.05) | 8(3.60) | 1(0.45) | 1(0.45) | 1(0.45) | 0(0.00) | 2(0.90) | 2(0.90) | 0(0.00) | 0(0.00) |
| Eating problems | 49(25.26) | 44(22.68) | 38(19.59) | 15(7.73) | 14(7.22) | 8(4.12) | 4(2.06) | 18(9.28) | 2(1.03) | 0(0.00) | 1(0.52) | 1(0.52) | 0(0.00) | 0(0.00) |
| Reflux | 63(26.58) | 59(24.89) | 33(13.92) | 25(10.55) | 17(7.17) | 7(2.95) | 13(5.49) | 13(5.49) | 2(0.84) | 2(0.84) | 0(0.00) | 1(0.42) | 1(0.42) | 1(0.42) |
| Odynophagia | 32(20.38) | 33(21.02) | 24(15.29) | 17(10.83) | 11(7.01) | 11(7.01) | 9(5.73) | 11(7.01) | 2(1.27) | 2(1.27) | 0(0.00) | 3(1.91) | 0(0.00) | 2(1.27) |
| Trouble swallowing saliva | 20(13.99) | 25(17.48) | 26(18.18) | 13(9.09) | 12(8.39) | 8(5.59) | 19(13.29) | 12(8.39) | 2(1.40) | 2(1.40) | 0(0.00) | 2(1.40) | 1(0.70) | 1(0.70) |

**Supplement table 5.** Cont.

| domains | Follow-up times | | | | | | | | | | | | | |
| --- | --- | --- | --- | --- | --- | --- | --- | --- | --- | --- | --- | --- | --- | --- |
|  | 1st | 2nd | 3rd | 4th | 5th | 6th | 7th | 8th | 9th | 10th | 11th | 12th | 13th | 14th |
| Choking when swallowing | 31(20.53) | 26(17.22) | 24(15.89) | 12(7.95) | 12(7.95) | 18(11.92) | 10(6.62) | 11(7.28) | 2(1.32) | 3(1.99) | 0(0.00) | 1(0.66) | 0(0.00) | 1(0.66) |
| Dry mouth | 26(17.69) | 23(15.65) | 27(18.37) | 13(8.84) | 13(8.84) | 6(4.08) | 8(5.44) | 14(9.52) | 3(2.04) | 4(2.72) | 3(2.04) | 2(1.36) | 2(1.36) | 3(2.04) |
| Trouble with taste | 20(18.35) | 12(11.01) | 18(16.51) | 11(10.09) | 12(11.01) | 8(7.34) | 8(7.34) | 13(11.93) | 0(0.00) | 2(1.83) | 1(0.92) | 4(3.67) | 0(0.00) | 0(0.00) |
| Coughing | 41(29.29) | 18(12.86) | 20(14.29) | 11(7.86) | 19(13.57) | 10(7.14) | 6(4.29) | 11(7.86) | 2(1.43) | 1(0.71) | 0(0.00) | 1(0.71) | 0(0.00) | 0(0.00) |
| Speech problems | 26(20.00) | 27(20.77) | 19(14.62) | 13(10.00) | 9(6.92) | 5(3.85) | 10(7.69) | 11(8.46) | 4(3.08) | 2(1.54) | 2(1.54) | 2(1.54) | 0(0.00) | 0(0.00) |

**Supplement table 6.** Association between types of tea consumption and EORTC QLQ-C30/EORTC QLQ-OES18 scales.

| Domain/scale |  | Non-fermented tea vs. No tea | | Semi-fermentation tea vs. No tea | |  | fully fermented tea vs. No tea | | Other tea vs. No tea | |
| --- | --- | --- | --- | --- | --- | --- | --- | --- | --- | --- |
|  |  | HR (95% CI) ^*^ | *P* | HR (95% CI) ^*^ | *P* |  | HR (95% CI) ^*^ | *P* | HR (95% CI) ^*^ | *P* |
| QLQ-C30 |  |  |  |  |  |  |  |  |  |  |
| Global health status/QOL |  | 0.729(0.479-1.109) | 0.140 | 0.771(0.555-1.073) | 0.123 |  | 1.098(0.659-1.830) | 0.720 | 0.664(0.335-1.317) | 0.242 |
| Physical functioning |  | 0.728(0.490-1.080) | 0.114 | 0.724(0.525-0.998) | 0.049 |  | 0.758(0.455-1.262) | 0.287 | 0.634(0.318-1.261) | 0.194 |
| Role functioning |  | 0.723(0.459-1.140) | 0.162 | 0.729(0.506-1.050) | 0.090 |  | 0.851(0.495-1.464) | 0.561 | 0.651(0.300-1.416) | 0.279 |
| Emotional functioning |  | 0.863(0.551-1.353) | 0.521 | 0.675(0.456-0.999) | 0.050 |  | 1.046(0.605-1.807) | 0.873 | 1.272(0.656-2.468) | 0.476 |
| Cognitive functioning |  | 0.654(0.395-1.082) | 0.098 | 0.798(0.532-1.196) | 0.274 |  | 1.048(0.581-1.891) | 0.875 | 1.103(0.530-2.296) | 0.794 |
| Social functioning |  | 0.957(0.615-1.490) | 0.847 | 1.106(0.768-1.592) | 0.587 |  | 1.017(0.552-1.875) | 0.956 | 0.828(0.379-1.810) | 0.637 |
| Fatigue |  | 0.739(0.470-1.161) | 0.189 | 0.791(0.553-1.130) | 0.198 |  | 1.189(0.699-2.022) | 0.524 | 1.090(0.560-2.124) | 0.800 |
| Nausea/vomiting |  | 0.899(0.572-1.415) | 0.647 | 0.778(0.526-1.152) | 0.211 |  | 1.283(0.726-2.267) | 0.390 | 0.405(0.148-1.110) | 0.079 |
| Pain |  | 0.666(0.409-1.086) | 0.103 | 0.992(0.681-1.444) | 0.966 |  | 1.132(0.642-1.994) | 0.669 | 0.863(0.397-1.879) | 0.711 |
| Dyspnea |  | 0.789(0.493-1.263) | 0.324 | 0.813(0.547-1.208) | 0.306 |  | 0.934(0.521-1.674) | 0.818 | 0.781(0.358-1.701) | 0.534 |
| Insomnia |  | 1.124(0.730-1.728) | 0.596 | 0.808(0.546-1.196) | 0.287 |  | 1.059(0.592-1.893) | 0.848 | 0.625(0.272-1.437) | 0.269 |
| Appetite loss |  | 0.753(0.475-1.195) | 0.229 | 0.972(0.668-1.417) | 0.884 |  | 1.156(0.657-2.032) | 0.615 | 0.485(0.196-1.200) | 0.117 |
| Constipation |  | 0.721(0.416-1.250) | 0.244 | 0.600(0.365-0.988) | 0.045 |  | 1.141(0.602-2.163) | 0.685 | 0.768(0.306-1.929) | 0.574 |

**Supplement table 6.** Cont.

| Domain/scale |  | Non-fermented tea vs. No tea | | Semi-fermentation tea vs. No tea | |  | fully fermented tea vs. No tea | | Other tea vs. No tea | |
| --- | --- | --- | --- | --- | --- | --- | --- | --- | --- | --- |
|  |  | HR (95% CI) ^*^ | *P* | HR (95% CI) ^*^ | *P* |  | HR (95% CI) ^*^ | *P* | HR (95% CI) ^*^ | *P* |
| Diarrhea |  | 0.968(0.616-1.523) | 0.889 | 0.774(0.514-1.167) | 0.222 |  | 1.035(0.563-1.903) | 0.911 | 0.494(0.199-1.229) | 0.129 |
| QLQ-QES18 |  |  |  |  |  |  |  |  |  |  |
| Dysphagia |  | 1.006(0.658-1.537) | 0.978 | 0.847(0.594-1.208) | 0.360 |  | 1.238(0.731-2.099) | 0.427 | 1.499(0.799-2.813) | 0.207 |
| Eating problems |  | 0.584(0.361-0.944) | 0.028 | 0.733(0.506-1.062) | 0.101 |  | 0.769(0.431-1.374) | 0.375 | 1.085(0.540-2.180) | 0.819 |
| Reflux |  | 0.934(0.629-1.387) | 0.735 | 1.054(0.763-1.457) | 0.748 |  | 1.016(0.594-1.739) | 0.953 | 0.553(0.268-1.143) | 0.110 |
| Odynophagia |  | 0.634(0.387-1.038) | 0.070 | 0.775(0.515-1.165) | 0.221 |  | 0.950(0.502-1.796) | 0.874 | 0.176(0.043-0.720) | 0.016 |
| Trouble swallowing saliva |  | 0.553(0.321-0.950) | 0.032 | 0.621(0.397-0.971) | 0.037 |  | 1.022(0.553-1.888) | 0.945 | 0.327(0.103-1.044) | 0.059 |
| Choking when swallowing |  | 0.487(0.279-0.853) | 0.012 | 0.858(0.570-0.853) | 0.461 |  | 1.680(0.944-2.990) | 0.078 | 0.407(0.148-1.119) | 0.081 |
| Dry mouth |  | 0.708(0.424-1.180) | 0.185 | 0.647(0.412-1.015) | 0.058 |  | 0.834(0.429-1.621) | 0.592 | 1.851(0.915-3.743) | 0.087 |
| Trouble with taste |  | 0.627(0.341-1.155) | 0.135 | 0.651(0.388-1.091) | 0.103 |  | 0.877(0.417-1.843) | 0.728 | 1.681(0.756-.0738) | 0.203 |
| Coughing |  | 0.546(0.312-0.959) | 0.035 | 0.634(0.406-0.988) | 0.044 |  | 1.099(0.582-2.078) | 0.771 | 0.291(0.091-0.928) | 0.037 |
| Speech problems |  | 0.490(0.269-0.893) | 0.020 | 0.726(0.465-1.135) | 0.160 |  | 0.909(0.467-1.768) | 0.778 | 0.300(0.094-0.959) | 0.042 |

^*^Controlling for demographic and clinical characteristics variables: age, income, Chemotherapy and TNM stage.

**Supplement table 7**. 65 male ESCC patients who did not drink tea preoperatively, and the EORTC QLQ -C30/EORTC QLQ -OES18 EORTC scale for both non-tea and tea drinking groups postoperatively to determine the clinically meaningful time to deterioration

|  |  | Time to deterioration [M (IQR)], n=65 | | | | | | |
| --- | --- | --- | --- | --- | --- | --- | --- | --- |
| Domain/scale |  | Non-tea-drinking |  | Tea-drinking |  | Z | *P* | |
| **QLQ-C30** |  |  |  |  |  |  |  |  |
| Global health status/QOL |  | 13.13(5.22，28.88) |  | 18.92(6.41，35.20) |  | -0.766 |  | 0.444 |
| Functional scales |  |  |  |  |  |  |  |  |
| Physical functioning |  | 15.67(7.98，22.67) |  | 23.26(7.20，27.21) |  | -1.245 |  | 0.213 |
| Role functioning |  | 21.13(12.32，34.17) |  | 13.55(8.14，41.09) |  | -0.734 |  | 0.463 |
| Emotional functioning |  | 22.57(10.12，37.49) |  | 24.31(9.84，51.36) |  | -0.351 |  | 0.726 |
| Cognitive functioning |  | 22.67(13.96，36.76) |  | 35.73(14.78，59.91) |  | -1.029 |  | 0.303 |
| Social functioning |  | 22.37（15.34,31.54） |  | 9.38(6.93，30.76) |  | -1.931 |  | 0.053 |
| Symptom scales |  |  |  |  |  |  |  |  |
| Fatigue |  | 13.04(6.44，24.97) |  | 19.50(6.90，43.10) |  | -1.189 |  | 0.234 |
| Nausea/vomiting |  | 17.54(12.45，29.57) |  | 27.89(21.40，33.67) |  | -1.875 |  | 0.061 |
| Pain |  | 17.54(12.85，32.00) |  | 28.27(6.93，43.43) |  | -0.191 |  | 0.848 |
| Dyspnea |  | 22.67(12.65，32.66) |  | 37.62(14.78，62.51) |  | -1.404 |  | 0.16 |
| Insomnia |  | 22.77(12.45，48.07) |  | 27.93(20.39，62.05) |  | -0.814 |  | 0.416 |
| Appetite loss |  | 17.45(9.13，37.49) |  | 24.97(12.61，44.66) |  | -0.75 |  | 0.453 |
| Constipation |  | 23.75(16.62，31.38) |  | 31.20(20.54，62.56) |  | -1.173 |  | 0.241 |
| Diarrhea |  | 22.67(12.65，36.14) |  | 33.86(22.39，62.51) |  | -1.947 |  | 0.052 |
| **QLQ-QES18** |  |  |  |  |  |  |  |  |
| General symptom scales |  |  |  |  |  |  |  |  |
| Dysphagia |  | 14.62(6.41，24.34) |  | 23.26(13.63，53.40) |  | -1.891 |  | 0.059 |
| Eating problems |  | 15.77(9.07，24.97) |  | 19.43(9.13，34.29) |  | -0.942 |  | 0.346 |
| Reflux |  | 16.59(10.09，22.67) |  | 10.96(7.01，39.87) |  | -0.431 |  | 0.667 |
| Odynophagia |  | 22.37(14.88，29.90) |  | 28.83(8.20，53.31) |  | -0.622 |  | 0.534 |
| General symptom items |  |  |  |  |  |  |  |  |
| Trouble swallowing saliva |  | 24.34(16.26，38.18) |  | 32.85(17.55，62.51) |  | -0.894 |  | 0.372 |
| Choking when swallowing |  | 24.97(16.26，36.18 |  | 30.54(17.55，53.31) |  | -0.790 |  | 0.430 |
| Dry mouth |  | 24.34(14.62，42.74) |  | 51.58(14.78，62.56) |  | -1.197 |  | 0.231 |
| Trouble with taste |  | 29.08(16.66，44.04) |  | 34.35(16.76，64.10) |  | -0.67 |  | 0.503 |
| Coughing |  | 22.57(11.47，36.76) |  | 40.94(21.77，64.10) |  | -2.12 |  | 0.034 |
| Speech problems |  | 24.97(15.93，53.22） |  | 24.07(8.14，62.05) |  | -0.112 |  | 0.911 |

**Supplement table 8.** Association between tea consumption and EORTC QLQ-C30/EORTC QLQ-OES18 scales in 65 male ESCC patients.

| Domain/scale |  | Univariate | | |  | Multivariate | | |
| --- | --- | --- | --- | --- | --- | --- | --- | --- |
|  |  | HR (95% CI) |  | P value |  | HR (95% CI) * |  | P value |
| QLQ-C30 |  |  |  |  |  |  |  |  |
| Global health status/QOL |  | 0.944(0.481-1.852) |  | 0.866 |  | 0.978(0.488-1.959) |  | 0.949 |
| Physical functioning |  | 0.798(0.415-1.533) |  | 0.497 |  | 0.729(0.366-1.450) |  | 0.367 |
| Role functioning |  | 1.099(0.545-2.218) |  | 0.791 |  | 1.179(0.566-2.455) |  | 0.660 |
| Emotional functioning |  | 1.017(0.481-2.150) |  | 0.966 |  | 1.021(0.470-2.212) |  | 0.957 |
| Cognitive functioning |  | 0.735(0.316-1.708) |  | 0.474 |  | 0.668(0.279-1.598) |  | 0.364 |
| Social functioning |  | 2.003(1.016-3.949) |  | 0.045 |  | 2.011(0.988-4.096) |  | 0.054 |
| Fatigue |  | 0.738(0.364-1.499) |  | 0.401 |  | 0.731(0.351-1.523) |  | 0.402 |
| Nausea/vomiting |  | 0.751(0.355-1.586) |  | 0.453 |  | 0.743(0.336-1.641) |  | 0.462 |
| Pain |  | 1.112(0.522-2.368) |  | 0.783 |  | 1.071(0.496-2.312) |  | 0.861 |
| Dyspnea |  | 0.551(0.241-1.261) |  | 0.158 |  | 0.504(0.216-1.176) |  | 0.113 |
| Insomnia |  | 0.876(0.398-1.925) |  | 0.741 |  | 0.786(0.349-1.767) |  | 0.560 |
| Appetite loss |  | 0.916(0.435-1.927) |  | 0.817 |  | 0.953(0.443-2.048) |  | 0.902 |
| Constipation |  | 0.802(0.342-1.881) |  | 0.612 |  | 0.715(0.297-1.719) |  | 0.453 |
| Diarrhea |  | 0.563(0.232-1.371) |  | 0.206 |  | 0.507(0.203-1.270) |  | 0.147 |
| QLQ-QES18 |  |  |  |  |  |  |  |  |
| Dysphagia |  | 0.552(0.257-1.189) |  | 0.129 |  | 0.558(0.245-1.268) |  | 0.164 |
| Eating problems |  | 0.812(0.403-1.636) |  | 0.560 |  | 0.856(0.416-1.758) |  | 0.671 |
| Reflux |  | 0.955(0.475-1.918) |  | 0.896 |  | 0.952(0.464-1.955) |  | 0.894 |
| Odynophagia |  | 0.951(0.446-2.026) |  | 0.896 |  | 0.905(0.406-2.019) |  | 0.808 |
| Trouble swallowing saliva |  | 0.606(0.232-1.586) |  | 0.308 |  | 0.481(0.174-1.331) |  | 0.159 |
| Choking when swallowing |  | 0.940(0.403-2.194) |  | 0.886 |  | 0.912(0.379-2.193) |  | 0.837 |
| Dry mouth |  | 0.897(0.380-2.118) |  | 0.804 |  | 0.783(0.319-1.919) |  | 0.593 |
| Trouble with taste |  | 1.091(0.424-2.810) |  | 0.856 |  | 1.002(0.365-2.753) |  | 0.997 |
| Coughing |  | 0.616(0.251-1.516) |  | 0.292 |  | 0.568(0.226-1.431) |  | 0.230 |
| Speech problems |  | 1.141(0.483-2.693) |  | 0.763 |  | 1.074(0.438-2.632) |  | 0.877 |

* Adjusted for age, income, chemotherapy and TNM stage

**Supplement table 9.** 225 male ESCC patients who consumed tea preoperatively, time to clinically significant deterioration determined by the EORTC QLQ -C30/EORTC QLQ -OES18 preoperative EORTC scale in both the no-tea and tea groups postoperatively.

|  |  | Time to deterioration [M (IQR)], n=65 | | | | | | | | |  |
| --- | --- | --- | --- | --- | --- | --- | --- | --- | --- | --- | --- |
| Domain/scale |  | Non-tea-drinking |  | Tea-drinking | | Z | | *P* | | |  |
| **QLQ-C30** |  |  |  |  |  | |  | |  |  | |
| Global health status/QOL |  | 11.86(5.39，21.83) |  | 18.00(6.31，38.90) |  | | -2.368 | |  | 0.018 | |
| Functional scales |  |  |  |  |  | |  | |  |  | |
| Physical functioning |  | 11.93(5.39，18.49) |  | 15.21(7.34，33.40) |  | | -2.771 | |  | 0.006 | |
| Role functioning |  | 15.85(9.03，27.36) |  | 22.69(10.68，47.75) |  | | -2.522 | |  | 0.012 | |
| Emotional functioning |  | 17.22(10.66，29.72) |  | 24.97(14.74，45.09) |  | | -2.878 | |  | 0.004 | |
| Cognitive functioning |  | 19.32(11.91，36.58） |  | 24.62(14.66，51.93) |  | | -2.478 | |  | 0.013 | |
| Social functioning |  | 18.51(9.37，29.00） |  | 22.78(10.37，40.31) |  | | -1.079 | |  | 0.280 | |
| Symptom scales |  |  |  |  |  | |  | |  |  | |
| Fatigue |  | 16.54(8.40，27.00) |  | 19.50(6.90，43.10) |  | | -1.450 | |  | 0.147 | |
| Nausea/vomiting |  | 17.48(9.49，29.72) |  | 27.89(21.40，33.67) |  | | -1.868 | |  | 0.062 | |
| Pain |  | 19.71(9.49，29.72) |  | 28.27(6.93，43.43) |  | | -1.255 | |  | 0.210 | |
| Dyspnea |  | 20.40(10.87，34.74) |  | 37.62(14.78，62.51) |  | | -1.764 | |  | 0.078 | |
| Insomnia |  | 17.77(8.99，29.89) |  | 27.93(20.39，62.05) |  | | -2.232 | |  | 0.026 | |
| Appetite loss |  | 16.92(10.14，29.13) |  | 24.97(12.61，44.66) |  | | -2.341 | |  | 0.019 | |
| Constipation |  | 24.97(14.19，44.42) |  | 31.20(20.54，62.56) |  | | -2.494 | |  | 0.013 | |
| Diarrhea |  | 19.29(10.64，29.60) |  | 33.86(22.39，62.51) |  | | -1.735 | |  | 0.083 | |
| **QLQ-QES18** |  |  |  |  |  | |  | |  |  | |
| General symptom scales |  |  |  |  |  | |  | |  |  | |
| Dysphagia |  | 14.78(6.50，20.85) |  | 13.80(6.72，21.09) |  | | -0.162 | |  | 0.871 | |
| Eating problems |  | 15.79(8.38，25.46) |  | 22.36(9.45，43.88) |  | | -2.496 | |  | 0.013 | |
| Reflux |  | 13.08(7.54，26.04) |  | 15.18(7.66，29.91) |  | | -1.164 | |  | 0.244 | |
| Odynophagia |  | 18.38(10.66，29.72) |  | 26.89(15.84，52.67) |  | | -3.098 | |  | 0.002 | |
| General symptom items |  |  |  |  |  | |  | |  |  | |
| Trouble swallowing saliva |  | 20.58(11.81，31.71) |  | 30.75(19.22，53.45) |  | | -3.824 | |  | P<0,001 | |
| Choking when swallowing |  | 20.11(9.80，29.79) |  | 27.04(14.60，53.45) |  | | -2.971 | |  | 0.003 | |
| Dry mouth |  | 21.90(13.18，37.23) |  | 31.18(17.15，57.20) |  | | -2.907 | |  | 0.004 | |
| Trouble with taste |  | 23.29(16.20，39.56) |  | 33.13(21.00，59.73) |  | | -3.235 | |  | 0.001 | |
| Coughing |  | 20.73(9.80，29.79) |  | 26.78(16.62，54.78) |  | | -3.395 | |  | 0.001 | |
| Speech problems |  | 20.40(11.47，36.62) |  | 37.16(19.43，55.93) |  | | -4.085 | |  | P<0,001 | |

**Supplement table 10.** Association between tea consumption and EORTC QLQ-C30/EORTC QLQ-OES18 scales in 225 male ESCC patients.

| Domain/scale |  | Univariate | | |  | Multivariate | | |
| --- | --- | --- | --- | --- | --- | --- | --- | --- |
|  |  | HR (95% CI) |  | P value |  | HR (95% CI) * |  | P value |
| QLQ-C30 |  |  |  |  |  |  |  |  |
| Global health status/QOL |  | 0.730(0.547-0.975) |  | 0.033 |  | 0.725(0.541-0.972) |  | 0.032 |
| Physical functioning |  | 0.688(0.519-0.912) |  | 0.009 |  | 0.689(0.518-0.917) |  | 0.010 |
| Role functioning |  | 0.650(0.474-0.890) |  | 0.007 |  | 0.678(0.493-0.933) |  | 0.017 |
| Emotional functioning |  | 0.732(0.529-1.012) |  | 0.059 |  | 0.750(0.541-1.040) |  | 0.084 |
| Cognitive functioning |  | 0.792(0.560-1.122) |  | 0.189 |  | 0.807(0.568-1.145) |  | 0.229 |
| Social functioning |  | 0.926(0.667-1.286) |  | 0.646 |  | 0.938(0.673-1.308) |  | 0.706 |
| Fatigue |  | 0.940(0.685-1.289) |  | 0.700 |  | 0.930(0.677-1.279) |  | 0.657 |
| Nausea/vomiting |  | 0.841(0.599-1.181) |  | 0.317 |  | 0.860(0.610-1.211) |  | 0.387 |
| Pain |  | 0.842(0.604-1.175) |  | 0.312 |  | 0.837(0.598-1.170) |  | 0.298 |
| Dyspnea |  | 0.866(0.616-1.217) |  | 0.406 |  | 0.900(0.639-1.258) |  | 0.547 |
| Insomnia |  | 0.850(0.611-1.182) |  | 0.335 |  | 0.862(0.618-1.202) |  | 0.382 |
| Appetite loss |  | 0.831(0.595-1.161) |  | 0.279 |  | 0.854(0.608-1.199) |  | 0.361 |
| Constipation |  | 0.734(0.489-1.101 |  | 0.135 |  | 0.754(0.501-1.134) |  | 0.175 |
| Diarrhea |  | 0.902(0.639-1.274) |  | 0.559 |  | 0.878(0.619-1.243) |  | 0.463 |
| QLQ-QES18 |  |  |  |  |  |  |  |  |
| Dysphagia |  | 0.957(0.712-1.285) |  | 0.768 |  | 1.038(0.766-1.408) |  | 0.809 |
| Eating problems |  | 0.716(0.516-0.993) |  | 0.045 |  | 0.701(0.502-0.980) |  | 0.037 |
| Reflux |  | 0.938(0.704-1.251) |  | 0.665 |  | 0.948(0.710-1.268) |  | 0.721 |
| Odynophagia |  | 0.667(0.463-0.961) |  | 0.030 |  | 0.679(0.469-0.983) |  | 0.040 |
| Trouble swallowing saliva |  | 0.578(0.397-0.842) |  | 0.004 |  | 0.596(0.407-0.872) |  | 0.008 |
| Choking when swallowing |  | 0.679(0.473-0.975) |  | 0.036 |  | 0.700(0.486-1.010) |  | 0.056 |
| Dry mouth |  | 0.712(0.493-1.028) |  | 0.070 |  | 0.741(0.511-1.073) |  | 0.113 |
| Trouble with taste |  | 0.674(0.441-1.031) |  | 0.069 |  | 0.672(0.437-1.033) |  | 0.070 |
| Coughing |  | 0.603(0.412-0.881) |  | 0.009 |  | 0.615(0.417-0.907) |  | 0.014 |
| Speech problems |  | 0.555(0.374-0.825) |  | 0.004 |  | 0.556(0.372-0.830) |  | 0.004 |

* Adjusted for age, income, chemotherapy and TNM stage
